# Glances at Foreign Asylums

**Published:** 1890-04-26

**Authors:** 


### Glances at Foreign Asylums.

[Annual Reports, Pamphlets, &c., should be addressed to The Editor, The Lodge, Porchester Square, London, W.]

### BLOOMINGDALE ASYLUM, NEW YORK.

This asylum has an average of three hundred patients in residence, and to look after these there are four regular medical officers and three clinical assistants. When measured by English standards this is a very high staff, and it is to be hoped and expected that the patients are well looked after in a medical point of view, and that much clinical work and investigation of disease are carried out.

There were during the year 187 admissions, and 52 were discharged recovered; 38 died.

It is worthy of note that among the patients was one who had recovered from thirty-two attacks of insanity, and Dr. Nichols thinks that these were separate seizures, and not periodical explosions of a substantially continuous disorder.

During part of the year the asylum was crowded on the women's side, and cases had to be refused admission. We often notice in our perusal of American reports that the same plethora exists in the asylums of the United States as in those of England.

### QUEBEC LUNATIC ASYLUM.

The report for 1888 opens with a sketch of the life of Dr. Roy, who was one of the directors and proprietors of the asylum. Apart from this little bit of biography, doubtless interesting enough to some of the Canadians, the report is a mere bundle of tables and statistics, from which we learn that there were 883 patients in the asylum; that, during the year, 104 were admitted, that 58 were discharged, and that 50 died. Of the admissions 87 spoke the French language, 16 English, and one some other language. There are only two resident physicians.

### DAYTON ASYLUM, OHIO.

The daily average number of patients in this asylum is about 550. The recoveries calculated on the admissions are 23·45 per cent., and the deaths stand at 9·7. The asylum is superintended by Dr. Calvin Pollock, and he has three assistant medical officers, one of whom is a lady. Dr. Pollock's report is much shorter than is usual with American superintendents; but we learn from it all we really want to know. We note that of the 51 deaths, no fewer than 9 were from consumption. This would be looked on as a high proportion in England, and would suggest that there is something wrong with the ventilation or the diet.

### SOUTHERN HOSPITAL FOR THE INSANE, ILLINOIS.

This may be described as a report of unusual interest. The asylum is of average size, for we see that the year closed with 630 patients in residence. There were 160 discharged during the year, and 52 died.

The open-door system has been tried successfully in part of the male side, and it is now being extended to the female wards with satisfactory results so far. We note that the electric light is in use.

Dr. Wardner emphasises what has many a time been stated on this side of the Atlantic—namely, that occupation is one of the best therapeutic agents in the treatment of insanity, especially in the convalescent stages. There are several remarks about the causation of the disease which we should like to see widely circulated among the laity, and we regret our inability to quote them here. We can endorse the following: "The development of a human being into a well-formed and well-balanced individual, in whom the habits, impulses, and intellect are so well formed and adjusted as to produce a character solid and symmetrical, is one of the greatest and most desirable of achievements."

